# Abnormal visual representations associated with confusion of perceived facial expression in schizophrenia with social anxiety disorder

**DOI:** 10.1038/s41537-020-00116-1

**Published:** 2020-10-01

**Authors:** Simon Faghel-Soubeyrand, Tania Lecomte, M. Archibaldo Bravo, Martin Lepage, Stéphane Potvin, Amal Abdel-Baki, Marie Villeneuve, Frédéric Gosselin

**Affiliations:** 1 Département de Psychologie, Université de Montréal, Montréal, Canada; 2 School of Psychology, University of Birmingham, Birmingham, United Kingdom; 3 Department of Psychiatry, McGill University, Montréal, Canada; 4 Départment de Psychiatrie, Université de Montréal, Montréal, Canada; 5 Centre hospitalier de l’Université de Montréal-Hôpital Notre-Dame, Montréal, Canada; 6 Institut universitaire en santé mentale de Montréal, Montréal, Canada

**Keywords:** Schizophrenia, Human behaviour, Emotion

## Abstract

Deficits in social functioning are especially severe amongst schizophrenia individuals with the prevalent comorbidity of social anxiety disorder (SZ&SAD). Yet, the mechanisms underlying the recognition of facial expression of emotions—a hallmark of social cognition—are practically unexplored in SZ&SAD. Here, we aim to reveal the visual representations SZ&SAD (*n* = 16) and controls (*n* = 14) rely on for facial expression recognition. We ran a total of 30,000 trials of a facial expression categorization task with Bubbles, a data-driven technique. Results showed that SZ&SAD’s ability to categorize facial expression was impared compared to controls. More severe negative symptoms (flat affect, apathy, reduced social drive) was associated with more impaired emotion recognition ability, and with more biases in attributing neutral affect to faces. Higher social anxiety symptoms, on the other hand, was found to enhance the reaction speed to neutral and angry faces. Most importantly, Bubbles showed that these abnormalities could be explained by inefficient visual representations of emotions: compared to controls, SZ&SAD subjects relied less on fine facial cues (high spatial frequencies) and more on coarse facial cues (low spatial frequencies). SZ&SAD participants also never relied on the eye regions (only on the mouth) to categorize facial expressions. We discuss how possible interactions between early (low sensitivity to coarse information) and late stages of the visual system (overreliance on these coarse features) might disrupt SZ&SAD’s recognition of facial expressions. Our findings offer perceptual mechanisms through which comorbid SZ&SAD impairs crucial aspects of social cognition, as well as functional psychopathology.

## Introduction

Deficits in social cognition, notably in the recognition of facial expression of emotions, are one of the hallmark impairments in individuals with schizophrenia (SZ)^1^. Social cognition deficits are significantly related to a person’s level of functioning^2^, and emotion perception in particular is strongly and consistently associated with various domains of functioning, such as community functioning, social behaviors, social skills, and social problem solving^3^.

SZ is also associated with multiple comorbid disorders, including anxiety disorders and more specifically social anxiety disorder (SAD)^4,5^. Several studies have demonstrated that not only does SAD worsen the prognosis of SZ, but also impedes the overall social recovery of the individual^6^. Furthermore, individuals suffering from comorbid schizophrenia and social anxiety (SZ&SAD) have a higher rate of suicide attempts, a high prevalence of alcohol/substance abuse disorders, and worse quality of life compared to those with SZ without social anxiety^7^. Thus, considering the serious consequences of comorbid SZ&SAD, better understanding the mechanisms underlying their emotion recognition deficits is important.

To our knowledge, only two studies have examined emotion recognition and confusion patterns in SZ&SAD^8,9^, and they have led to contradictory results. The current study’s main objective is to resolve this conflict and to shed light on the perceptual mechanisms responsible for the categorization of facial expression of emotions in SZ&SAD. To do so, we used Bubbles^10^, a data-driven method, which enabled us to reveal the specific visual representations (i.e., the precise facial features and spatial frequency information) that individuals with SZ&SAD rely on to decode facial expression of emotions^11–13^. This also allowed us to measure SZ&SAD’s emotion recognition ability with robust psychophysical metrics, to describe their emotion confusion patterns, and to reveal any association between these facial-affect processing and their psychiatric symptoms. Given the absence of studies on the visual representations in individuals with SZ&SAD and even in SAD, the following paragraphs will focus on reviewing findings relevant to the categorization of facial expression of emotions in individuals with SZ.

In individuals on the SZ spectrum, the presence of facial expression of emotion categorization deficits is now well-established. In an attempt to explain these impairments, clinical and cognitive psychologists have recently proposed that the emotion perception abnormalities of SZ may emerge from over-attributing specific emotions to affective faces, specifically negative emotions such as anger, fear, or disgust^14–18^. Likewise, psychologists have speculated that socially anxious individuals should also show a bias for negative emotions because of their fear of being judged in a negative way by their peers^19,20^. Empirical studies in both clinical and non-clinical populations have revealed some associations between a bias for negative emotions and social anxiety traits^11,21–26^. As far as we know, the joint effect of SZ&SAD on categorization biases has only been explored in one study^9^. The authors observed that the presence of social anxiety in SZ resulted in patients having more difficulty recognizing neutral faces, confusing them with faces expressing other emotions, compared to those with SZ and no social anxiety.

Prior work using behavioral and brain imaging methods with SZ individuals has revealed low-level perceptual abnormalities that may be linked to these facial expression categorization impairments and biases. Indeed, individuals with SZ tend to have reduced contrast sensitivity to low spatial frequency (LSF; coarse visual information) gratings^27–29^ and reduced sensitivity to LSF-filtered faces^30–32^. These studies as well as others^33,34^ have promoted the idea of a magnocellular deficit in SZ^35^. Using similar methods, researchers have also proposed that deficiencies at a higher-level of the visual system could explain poor facial-affect processing in SZ. It is thought, for example, that top-down modulation processes such as the “coarse-to-fine” object/face-recognition process^36,37^ could be linked to the emotion processing impairments of SZ patients^38,39^. Arguing for deficits on such top-down processes, recent brain imaging studies^39^ have proposed that individuals with SZ may compensate for their poor processing of LSF (coarse) information with a visual strategy relying more on high spatial frequency (HSF, fine) information. Quite surprisingly, however, even though these past studies have speculated on the nature of the high-level internal representations SZ rely on in various face-recognition tasks, only two studies have looked directly at such visual representations in SZ^12,13^. These two studies found atypical visual representations in SZ (e.g., less reliance on the eye region; see also eye-tracking studies^40–43^), but no evidence for a bias toward HSF in SZ. Again, however, no one has ever explored the effect of clinically diagnosed social anxiety, nor the joint effect of SZ and clinical social anxiety on visual representations.

The objectives of the present study were thus the following: (1) to measure the general ability for facial expression recognition in individuals with SZ&SAD; (2) to examine categorization biases in this population and to explore their links with psychiatric symptoms; and (3) to reveal the visual memory representations underlying the facial expression of four basic emotions in SZ&SAD (joy, fear, anger, neutrality). To be clear, the focus of this study was not to demonstrate that SZ&SAD has a different etiology or pathophysiology than SZ. Rather, this study’s main objective was to examine social cognition in this particular comorbidity because, as we pointed out above, it is relatively frequent, it significantly worsens SZ prognosis and it has received little attention in the SZ literature.

## Results

**Fig. 1:**
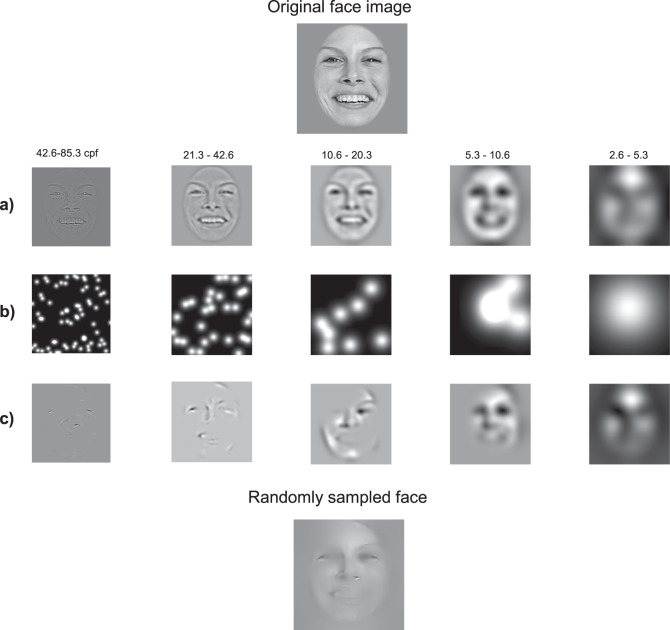
Creation of the “bubblized” stimuli. **a** An original face image was decomposed into five spatial frequency bands using a Laplacian transform. **b** Small Gaussian apertures (i.e., the bubbles) were then placed at random locations for each spatial frequency band separately. **c** Finally, the information revealed by the bubbles for each SF band was fused across the five frequency bands to produce one experimental stimulus (bottom image). The authors have obtained consent for publication of the face image depicted in this figure.

The Bubbles procedure is illustrated in Fig. 1. On each trial, a face expressing an emotion was randomly selected among a set of 40, and randomly sampled using Gaussian apertures (bubbles) in the 2D image plane and in five spatial frequency bands (for more details, see “Methods” section and ref. ^10^). The quantity of visual information (i.e., the number of bubbles) was adjusted throughout the task by the QUEST algorithm^44^ so that each subject maintained an accuracy of 75%. This quantity of visual information was our measure of face-emotion categorization performance.

### Emotion categorization performance

We first tested the hypothesis that individuals with comorbid SZ&SAD have impaired recognition of facial expression of emotions. One SZ&SAD participant was excluded from further analysis because of his poor performance (i.e., number of bubbles threshold >${\bar{X}}$ ± 3*σ*; *N*_SZ&SAD_ = 15). No control participants were excluded based on this criteria. The resulting total sample’s (i.e., SZ&SAD and controls) average number of bubbles to maintain an accuracy of 75% ranged from a low of 46 (i.e., best performance) to a high of 231 (poorest performance).

SZ&SAD performed worse—they required more bubbles—than neurotypicals to categorize facial expression of emotions (*M*_SZ&SAD_ = 130.3471, *M*_CTL_ = 90.3380; STDEV_SZ&SAD_ = 50.8760, STDEV_CTL_ = 34.3402, F(26, 1) = 5.75, *p* = 0.0239, Cohen’s *d* = 0.91) and were slower than controls (*F*_group_effect_(104, 1) = 18.8, *p* < 0.0001, Cohen’s *d* = 0.829; *F*_emotion_effect_(104, 3) = 4.37, *p* = 0.006; *F*_interaction_(104, 3) = 0.22, *p* > 0.3).

### Association between emotion categorization performance and clinical psychopathology

We then tested if this impairment in emotion recognition ability is associated with SZ&SAD’s clinical psychopathology. The more negative symptoms (blunted affect, apathy, and reduced social drive as assessed by the BPRS, see “Methods” section) a person with SZ&SAD displayed, the worse this person was at categorizing facial expression of emotions (*r*(14) = 0.5768, *p* = 0.0244). There was no association between reaction time and BPRS symptoms (all *ps* > 0.05). Social anxiety symptoms (as assessed by the total score of the Brief Social Phobia Scale (BSPS), see Method section) were not significantly linked significantly to categorization performance (*r*(14) = −0.42, *p* = 0.14); they were linked, however, to *faster* reaction times for both anger and neutral faces in participants with SZ&SAD (*r*_*anger*_(14) = −0.63, *p* = 0.01; *r*_*neutral*_ = −0.52, *p* = 0.04). More specific (post-hoc) tests were conducted on the association between the categorization speed of neutral and angry facial expressions and dimensions of social anxiety symptoms. Only the association between the avoidance dimension and the categorization speed of neutral faces reached statistical significance after Bonferroni-correction (*r*_*neutral*_(14) = *−*0.64*, p* = 0.0109, Bonferroni-corrected critical *p* = 0.0166).

### Association between emotion confusions and clinical psychopathology

**Fig. 2:**
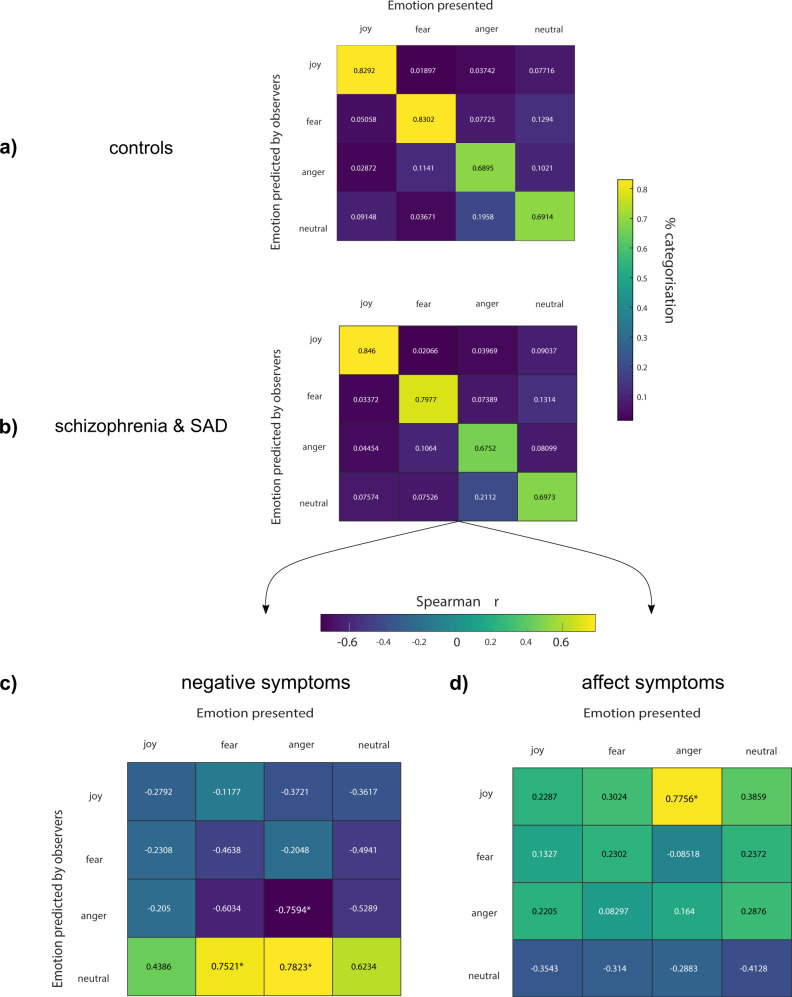
Association between facial expression confusions and clinical psychopathology. Confusion of facial expression of emotions in **a** controls (*n* = 14) and **b** SZ&SAD (*n* = 16). **c** Correlation between the SZ&SAD confusion matrix and clinical negative symptoms. **d** Correlation between the SZ&SAD confusion matrix and clinical affective symptoms.

Next, we explored the associations between specific emotion confusions (e.g., angry face labelled as fearful) and clinical psychopathology. The confusion matrix we derived for the SZ&SAD group (shown in Fig. 2a, second row) first revealed three significant frequent confusions during the categorization of facial expression of emotions: (1) neutral faces were often miscategorized as “fearful”, (2) angry faces were often miscategorized as “neutral”, and (3) fearful faces were often miscategorized as “angry” (Probability_Observed_ = 13.14 %, *χ*^2^ = 112.0896; Probability_Observed_ = 13.14 %, *χ*^2^ = 112.0896; Probability_Observed_ = 10.64 %, *χ*^2^ = 24.9799, Probability_Expected_ = 8.3 %, i.e., $\frac{{\left( {1 - {\rm{accuracyExpected}}} \right)}}{{\left( {n{\rm{Class}} - 1} \right)}}$, all *ps* < 0.003, the Bonferroni-corrected critical *p*). There were no significant local contrasts between confusions of SZ&SAD and controls.

The correlation between individual biases in the decoding of specific facial expressions and the four BPRS dimensions described earlier is shown in Fig. 2c, d. Congruent with our previous result, SZ&SAD individuals with more severe negative symptoms were more prone to miscategorize angry and fearful faces as “neutral” (*r*(14)_angerAsneutral_ = 0.7823; *r*(14)_fearAsneutral_ = 0.7521, *ps* < 0.003, Fig. 2c). We also found a negative correlation between negative symptoms and accurate anger categorization (*r*(14) = −0.7594, *p* < 0.003; Fig. 2c). Moreover, the higher an individual with SZ&SAD scored on the affect dimension (presence of depression, anxiety and guilt), the more this individual confused angry with joyful faces (*r*(14) = 0.7756, *p* < 0.003; Fig. 2d).

### Visual representations for accurate emotion categorization

**Fig. 3:**
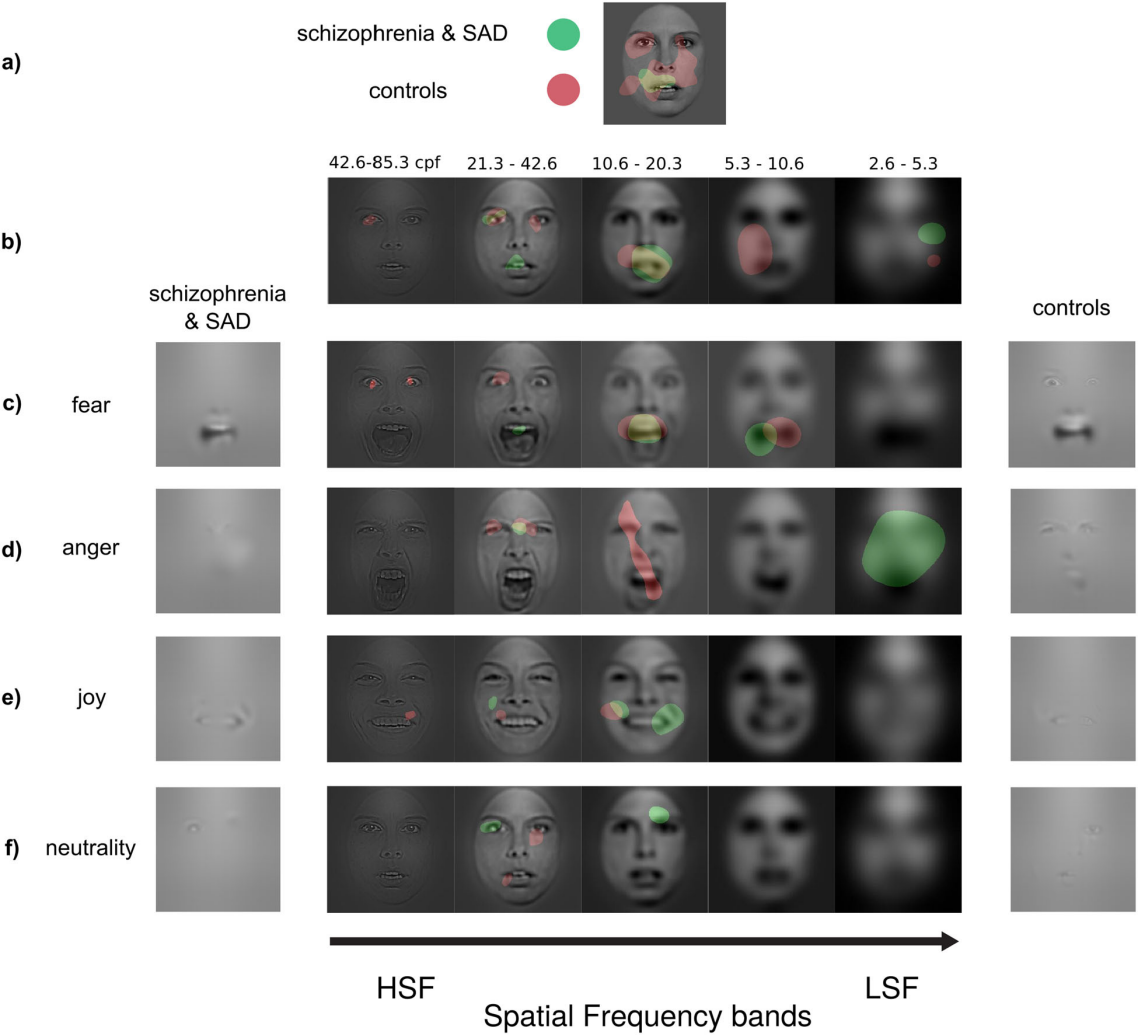
Visual representations of facial expressions in SZ with anxiety. Regions attaining statistical significance (*p* < 0.05, two-tailed) in the classification images obtained during the categorization of facial expression of emotions in SZ&SAD (green, *n* = 16) and controls (red, *n* = 14). **a** Classification images (CI) were pooled across participants from the same subject group, spatial frequency scales and emotions. They show the overall use of facial features during this task for SZ&SAD and controls. **b** CI were pooled across participants from the same subject group and emotions. They show the overall use of facial features at different spatial frequencies during emotion recognition for SZ&SAD and controls. **c**–**f** CI were pooled across participants from the same subject group. They show the use of features at different spatial frequencies for **c** fear, **d** anger, **e** happy and **f** neutral faces for SZ&SAD and controls. All facial features used above statistical threshold for each emotion are also displayed on the left (SZ&SAD) and right side (controls) to help interpretation. The authors have obtained consent for publication of the face images depicted in this figure.

Finally, we revealed the visual memory representations underlying the facial expressions of emotions in SZ&SAD using *Bubbles*. The classification images (CIs) of control participants show that they used the mouth and, to a lesser extent, the eyes (mostly the left eye) to categorize facial expression of emotions (Fig. 3a; see also ref. ^45^). SZ&SAD participants, however, only relied on the mouth region to categorize facial expressions.

We also examined the use of facial features at different spatial frequencies across emotions (see Fig. 3b). Individuals with SZ&SAD (green) represented emotion cues from the mouth at spatial frequencies (SF) between 10.6 and 42.6 cycles per face (cpf). For controls (red), their visual representation of eye emotion cues contained only high SF (>21.3 cpf), while the mouth region contained only lower spatial frequencies (20.3–5.3 cpf).

We also examined the use of different spatial frequencies across emotions and facial features. This revealed that SZ&SAD relied overall less on high spatial frequency face-emotion information compared to controls (42.6–85.3 cpf; *M*_SZ&SAD_ = 0.0741, *M*_CONTROL_ = 0.1414, *t*(27) = −3.228, *p* = 0.0033, Cohen’s *d* = −1.29). In contrast, SZ&SAD used low SF face-emotion information more than controls (2.6–5.3 cpf; *M*_SZ&SAD_ = 0.2369, *M*_CONTROL_ = 0.0722, *t*(27) = 2.4518, *p* = 0.0210, Cohen’s *d* = 0.9443; mid-range SFs showed no significant contrasts).

We finally examined the use of facial features at different spatial frequencies for all emotions (see Fig. 3c–f). Individuals with SZ&SAD mostly represented emotion cues from the mouth at spatial frequencies between 10.6 and 42.6 cpf. For controls, their visual representation of eye emotion cues contained only high SFs (>21.3 cpf), while the mouth region contained only lower spatial frequencies (20.3–5.3 cpf). More specifically, CIs for fear recognition showed that individuals with SZ&SAD did not rely on the typical bilateral eye regions in high SF content, whereas controls did (e.g., ref. ^45,46^). Similarly, CIs for anger recognition showed that SZ&SAD patients did not rely on eye cues in high SFs; they rather used a region between both eyes in mid-high SF (i.e., frown lines) to do so.

## Discussion

A better understanding of the mechanisms underlying emotion recognition in individuals with comorbid SZ&SAD is important considering both the prevalence and serious consequences of this additional diagnostic in SZ. Here, we used Bubbles, a data-driven psychophysical approach, and revealed the specific visual representations supporting facial expression of emotion recognition in SZ&SAD. We found, first, that SZ&SAD individuals have impaired recognition of facial expressions: They require more visual information and are slower than controls to recognize emotions at a 75% correct rate. This confirms and extends the evidence for emotion processing impairments in SZ patients^1^ to SZ&SAD patients. Second, we showed that more severe negative symptoms (blunted affect, apathy, and reduced social drive) were associated with poorer recognition of emotions expressed facially. This also aligns with previous findings on the effects of negative symptoms in SZ^32,41,47–50^ and generalizes them to SZ individuals with SAD. Then, we discovered that more severe negative symptoms specifically impact emotion perception through a bias for neutral emotions. Interestingly, affect symptoms (anxiety, depression, guilt, and somatic concern) were associated with a qualitatively different confusion pattern, i.e., with higher confusions of angry faces. Considering that impaired emotion perception is especially present in individuals with SZ suffering from flat affect^41,47^, our finding of face representation biases toward neutral affect suggest that the visual representations of emotions might not only be central to perception, but also to the expression of facial-affect in these individuals.

Another finding linking clinical psychopathology to emotion processing in individuals with SZ&SAD was that higher social anxiety (specifically avoidance) symptoms actually *enhanced* the speed at which they react to facial expressions of anger and neutral. This finding supports the idea of an important role of emotion valence in SZ&SAD’s emotion processing mechanisms. Indeed, socially phobic individuals have been described to have an abnormal—but not necessarily impaired—processing of negative emotions from social stimuli^26^. Faster processing of emotions of negative valence in these individuals is generally thought to arise from an early hypervigilance to threatening social stimuli^26,51^. While it is tempting to dissociate the respective effect of social anxiety (hypervigilance to negative emotions) and negative symptoms (poor representations biased toward neutral affect) in SZ&SAD, additional experimental studies will be needed to shed light on their respective effect on facial-affect processing in SZ.

Most importantly, we showed that these emotion recognition impairments in SZ&SAD’s could be explained by their inefficient visual representations of facial expression of emotions. Compared to typical controls, SZ&SAD relied less on fine facial cues (high SF) and more on coarse facial cues (LPSs). Several studies with SZ individuals have found disruption in the early stage (magnocellular) process of the vihual system, with, for example, impaired contrast sensitivity for LSF gratings (coarse information^27,29,52^). It has been proposed, as a consequence of this low-level processing, that SZ individuals should rely on high SFs more than controls for resolving visual tasks^39^. We clearly demonstrated that this is not the case. Instead, our results are in agreement with two other studies that examined the relative importance of coarse and fine information in the classification of objects^53^ and faces^54^, and found a bias for coarse information. However, we disagree with the conclusion reached by the researchers according to which this LSF bias refutates the magnocellular pathway deficits hypothesis of SZ. We rather believe that the impaired recognition of facial expression of emotions in SZ results from an interaction between lower- and higher-level processing mechanisms. In other words, we propose that it is the joint effect of (1) their poor sensitivity to low spatial frequencies in early stages of the visual system and (2) the LSF bias of their higher-level (facial-affect) visual representations that produce these impairments.

Another noteworthy finding is that SZ&SAD individuals never represented the eye regions (only on the mouth area) in their visual representations of facial expression of emotions. Lee et al.^12^ and Clark et al.^13^ used the Bubbles technique to evaluate the visual representations of SZ participants during facial emotion recognition tasks, and found, much like we did in SZ&SAD participants, that SZ participants relied less on the eye regions than control participants. Most eye-tracking results have also corroborated these *Bubbles* findings by showing that SZ individuals tend to gaze less frequently at the eyes of faces^40–43^ than controls (but see refs. ^38,55,56^). However, the SZ participants of Lee et al.^12^ did use the eye regions in mid-to-high SFs to categorize both fear and happy faces. Likewise, the SZ participants of Clark et al.^13^ utilized the eye regions to categorize angry faces across spatial frequencies. These findings indicate a somewhat impaired representation of eye cues in SZ. In contrast, our SZ&SAD group did not rely on the eye region at all for any of the emotions or at any SF scale tested in these previous studies. Such extreme impaired eye representation is also at odds with what was found, using a similar method, in (non-clinical) social anxiety participants^11^. Our results appear to be more in line with findings from eye-tracking studies in clinically diagnosed socially anxious individuals^57^. That being said, our lack of SZ-only and SAD-only (*clinically* diagnosed social anxiety) control groups precludes the dissociation between main effects of SZ and SAD and their interaction on facial expression representations. Future studies with these control groups will be needed to draw more definitive conclusions. Nonetheless, we found significant impairments in both social cognition and core visual feature extraction mechanisms in the SZ&SAD population. These deficits stress the need for more empirical work on this population, and for a systematic assessment of SAD comorbidity in samples of SZ individuals.

Interestingly, the underutilization of the eye regions in our SZ&SAD participants can be explained by a systematic *confusion* of the emotion cues in this area. Indeed, additional CI analysis on the facial information SZ&SAD relied on when they confused a face (i.e., fearful) with another emotion (i.e., anger) revealed that their confusion of these emotions emerged from their misinterpretation of cues from the eyes (see Supplementary Fig. 1). This mechanism might explain why anger and fear tend to be confused in individuals with SZ^15,17,18,32,58^ as well as in the present study. This confusion analysis also showed that emotion cues systematically misinterpreted by SZ&SAD were mainly present for negative valence emotions, which supports the idea of abnormal representations of emotions with negative valence in SZ&SAD.

Another weakness of this study is the sample size. Although it compares favorably with most Bubbles or reverse-correlation studies (which tend to have few subjects but many trials per subject), it is relatively small for a psychiatric-disorder study. We believe this work is still valuable because it is among the first to describe impairments in the recognition of facial expression of emotions in individuals with comorbid SZ&SAD. Assuredly, additional studies will be needed to draw more definitive conclusions. A systematic assessment of SAD in SZ samples might help to better understand the representations of facial expression of emotions in this population, if only by helping researchers recruit individuals with comorbid SZ&SAD. Finally, results might be tinted by our participants being mainly first-episode patients. We do not believe, however, that this can account for the effects shown here because impairments in facial expression recognition typically get worse after the first episode^59,60^. If anything, this suggests that our study might underestimate SZ&SAD’s effect on social cognition.

Finally, our finding that the visual representations of typical and SZ&SAD individuals differ may lead to a new kind of perceptual intervention for SZ&SAD individuals. Indeed, some of us recently devised a training procedure capable of inducing the use of specific facial information in typical participants; and showed that the induction of the visual representation of the best face recognizers in individuals with intermediate face-recognition abilities led to an enhancement of their face-recognition abilities^46,61,62^. We hope that by inducing the visual representations of typical individuals (i.e., eye emotion cues) in SZ&SAD with similar interventions will reduce their impairment to recognize facial expression of emotions and, in turn, help their social functioning.

## Methods

### Participants and procedure

A total of 30 participants were recruited for this study. The first group consisted of 16 individuals diagnosed with both a SZ spectrum disorder (SZ, *n* = 9; schizo-affective disorder, *n* = 7) and comorbid SAD. The second group was composed of 14 normal controls (see Table 1 for sociodemographic and clinical information). Clinical participants were referred and diagnosed by the attending psychiatrist (according to the DSM-5 criteria), by means of the first-episode psychosis clinic of the *Institut Universitaire en Santé Mentale de Montréal* (IUSMM), the *Centre Hospitalier de l’Université de Montréal* (*CHUM)* and the *Douglas institute Centre for Personalized Psychological Intervention for Psychosis*. At the time of recruitment, most patients (64.7%) had experienced a single psychotic episode. All clinical participants were medicated with atypical antipsychotics (mean dose in chlorpromazine equivalent was 299.9 mg/day), and were considered stabilized by the attending psychiatrist. The normal control group was composed of neurotypical individuals, i.e., individuals who did not have any psychological or neurological disorder. This study was approved by the Ethics and Research Committee of the IUSMM, CHUM, Douglas Institute and the ethics committee of the Université de Montréal, and informed (written) consent was obtained from all participants.

**Table 1:** Sociodemographic and clinical information of participants.

	SZ&SAD group (*n* = 16)	Control group (*n* = 14)	Group differences
Age	27.9 (4.4)	27.2 (4.4)	*p* = 0.590
Sex	43.8% women	33.3% women	*p* = 0.722
Years of education	12.9 (2.4)	16.8 (3.3)	*p* = 0.001
Age first consultation	20.9 (5.2)	–	–
No. hospitalizations	2.1 (2.1)	–	–
Duration of illness (years)	4.7 (4.1)	–	–
Brief Psychiatric Rating Scale			
Negative symptoms	9.6 (3.0)	–	–
Positive symptoms	9.6 (6.9)	–	–
Affect	12.8 (5.9)	–	–
Activity	9.8 (2.7)	–	–
Total score	41.8 (12.8)	–	–
Brief Social Phobia Scale			
Fear	16.6 (6.3)	–	–
Avoidance	16.5 (5.6)	–	–
Physiological	5.9 (3.3)	–	–
Total score	39.1 (12.2)	–	–

Mean score (standard deviation).

All participants were administered a sociodemographic survey, and performed the *Bubbles Facial Emotion Recognition Task* (henceforth called *Bubbles experiment*). In addition, clinical participants were evaluated with the following instruments: (1) *Structured Clinical Interview for DSM-5* (SCID^63^), which allowed to confirm patient diagnosis according to the DSM-5 criteria; (2) *Brief Psychiatric Rating Scale–Expanded Version* (BPRS^64,65^, which consists of a 24-item semi-structured interview that assesses the severity of psychiatric symptoms and which was delivered by trained-to-gold-standard graduate students. The following dimensions were used for analyses: negative symptoms, positive symptoms, affect, and activity; (3) BSPS^66^, which evaluates the severity of social anxiety and includes 18 self-report items answered on a five-point Likert scale (from 0 to 4). The scale has adequate psychometric properties^66^. The following subscales were generated: fear, avoidance, and physiological activation.

### Bubbles experiment

Forty grayscale face stimuli (256 × 256 pixels; ~5.72 × 5.72 deg of visual angle) from the STOIC database ([five females & five males] × four facial expressions^12,13,45,67^) were used as base faces. The emotions conveyed by these stimuli (joy, fear, anger, and neutrality) are expressed by trained actors and were selected to be highly recognizable.

To map which parts of expressive faces at different spatial scales were used by individuals with SZ&SAD and controls, we used the Bubbles method and an emotion categorization task (joy, fear, anger, neutrality). This sampling procedure is illustrated in Fig. 1 (for further details, see ref. ^10^). Briefly, faces were drawn randomly from the 40 exemplars as well as their mirror-flipped images, and randomly sampled using Gaussian apertures (bubbles) in the 2D image plane and in five spatial frequency bands. Spatial filtering was achieved with Laplacian pyramid transforms^68^. The quantity of visual information (i.e., the number of bubbles) was adjusted throughout the task by the QUEST algorithm^44^ so that each subject maintained an accuracy of 75%. This quantity of visual information was our measure of face-emotion categorization performance (see data analysis section). These stimuli were shown at the center of the screen and remained there until the subject pressed a key corresponding to one of the four possible emotions. Every participant completed 10 blocks of 100 trials each.

### Association between emotion categorization performance and clinical psychopathology

To associate emotion recognition processes to clinical psychopathology, individual psychophysical metrics within the SZ&SAD cohort (e.g., individual facial expression recognition performance thresholds) were Spearman-correlated with individual clinical scores (e.g., social anxiety trait score, as measured by the BSPS). Individual psychophysical performance thresholds for the emotion categorization task^69,70^ were computed from the best-fitted Gaussian cumulative distribution function (i.e., accuracy as a function of number of bubbles apertures across trials), and compared between SZ&SAD and controls with two-tailed unpaired *t* tests. Individual average reaction times for correct responses were computed for the four emotion categories, discarding outlier trials (trials with *Z* > 2.5, *Z* < −2.5 or <200 ms were rejected). Corrections for family-wise error rate (FWER) were applied within each group of post-hoc tests. Here, for example, Bonferroni corrections were applied when specifying the main effect between reaction times and social anxiety, dividing the critical *p* value by the number of subscales of social anxiety symptoms, i.e., 0.05/3 (0.0166).

### Confusions: association with clinical psychopathology

To reveal if SZ&SAD’s clinical psychopathology was associated with specific confusion patterns between emotions, a 4 × 4 (categorized emotion × presented emotion) confusion matrix was first derived for each individual and averaged across participants. Specifically, we computed the probability that each observer classified a given signal (e.g., an angry face) as a specific emotion (e.g., neutral), for each possible categorization and signal type. To know whether and which specific confusion patterns were associated with clinical psychopathology, we Spearman-correlated the between-individual variation in psychiatric score of interest (BRPS & BSPS scores) with every categorization x facial-affect probability pair (e.g., an angry face confused as a neutral one). We controlled for FWER using Bonferroni corrections: we divided the critical *p* value by 16—0.05/16 (0.0037)—the number of statistical tests against the null hypothesis (e.g., no relationship between negative symptoms and emotion confusions).

### Bubbles CIs

Our main goal was to reveal the visual memory representations of individuals with SZ&SAD when categorizing the facial expression of four basic emotions. Considering its use to explore complex memory representations, reverse-correlation techniques prescribes a high number of trial repetition per participant. In this study, thus, robust within-participants measures (1000 trials per individual, for a total of >30 000 trials) was preferred over a large sample size^71,72^. To reveal these visual representations, we performed multiple linear regressions on the location of the bubbles and z-scored response accuracies across trials, for each subject, spatial frequency band and emotion. Each of these regressions produces a plane of regression coefficients that we call a Classification Image (CI); it reveals parts or features of faces—in their specific spatial frequency band—that are systematically associated with a participant’s accurate emotion categorization. CIs were then summed across participants of each group per emotion and frequency band, smoothed with a Gaussian kernel of 15 pixels of standard deviation, and transformed into *Z* scores. Cluster tests^73^ (search region = 65,536 pixels; arbitrary *z*-score threshold = 2.7; cluster size statistical threshold = 277 pixels; *p* < 0.05, two-tailed) were used to assess the statistical significance of the resulting group CIs. The Cluster test corrects for FWER while taking into account the spatial correlation that is inherent to the *Bubbles* method. To specifically test whether SZ&SAD relied more on high SF, we contrasted (unpaired *t* test), between SZ&SAD and controls, the individual average *z*-scores contained in the face area for each of the five frequency bands.

### Reporting summary

Further information on research design is available in the Nature Research Reporting Summary linked to this article.

## Supplementary information

reporting summary

supplementary material

## Data Availability

The data that support the findings of this study are available from the corresponding author upon reasonable request.

## Code availability

The MATLAB code that supports the findings of this study are available from the corresponding author upon reasonable request.
