# Supplementary material for: Abnormal visual representations associated with confusion of perceived facial expression in schizophrenia with social anxiety disorder
Source: NPJ Schizophr. 2020 Oct 1;6:28. doi: 10.1038/s41537-020-00116-1 (PMC7529755; doi:10.1038/s41537-020-00116-1)
Supplement: Supplementary file 2 — supplementary material [file 41537_2020_116_MOESM2_ESM.pdf]

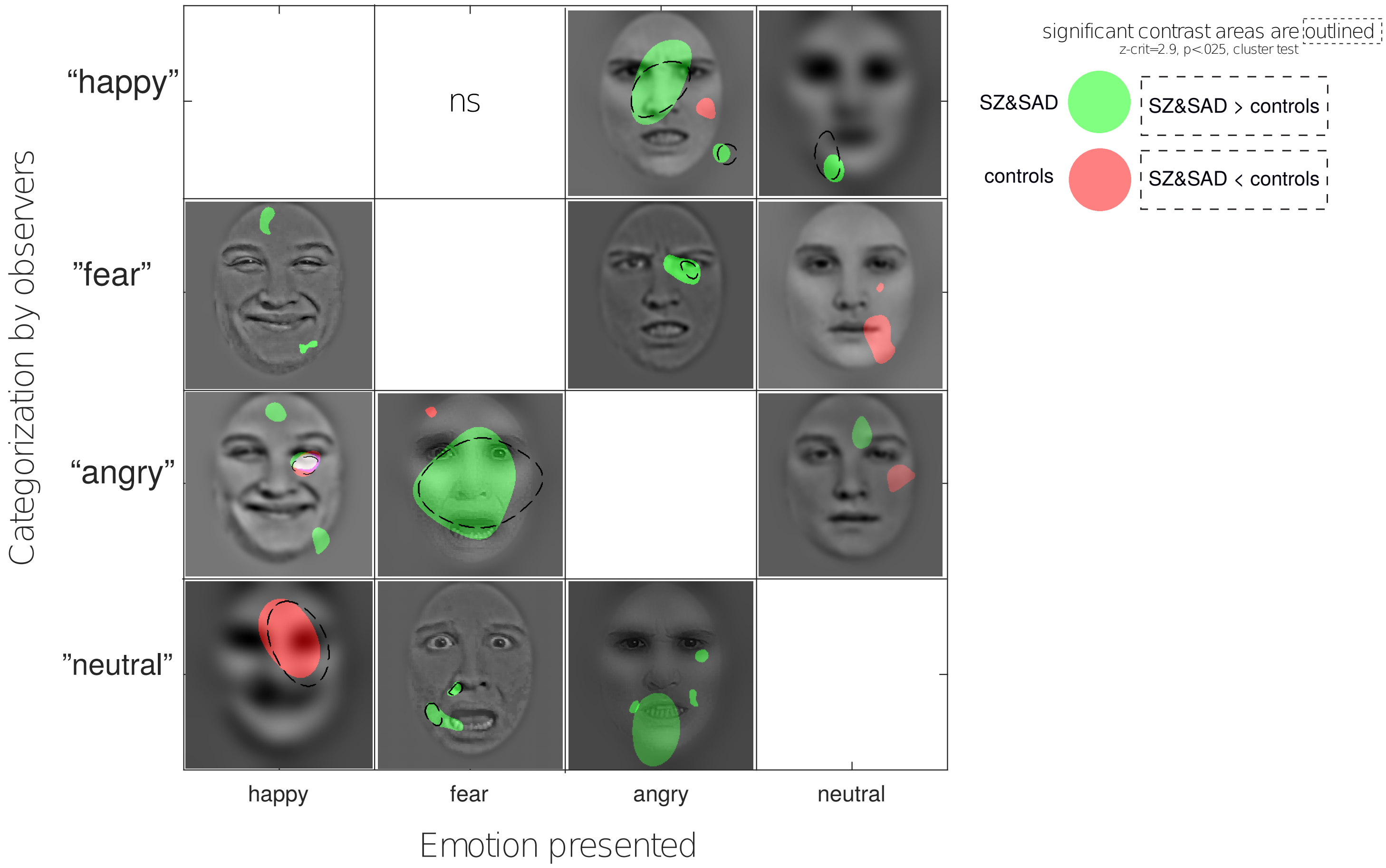

Supplementary Figure 1. Facial cues driving emotion mis-attributions in SZ&SAD and controls
Contrast between groups is shown in dotted lines (for details, see the Classification images section).
The authors have obtained consent for publication of the face images depicted in this figure.
